# Multivariate evaluation method for the detection of pest infestations on plants via VOC analysis using gas chromatography mass spectrometry

**DOI:** 10.1038/s41598-025-11607-5

**Published:** 2025-07-16

**Authors:** Sarah Vermeeren, Markus Witzler, Ramona Makarow, Carsten Engelhard, Peter Kaul

**Affiliations:** 1 Institute for Safety and Security Research, Hochschule Bonn-Rhein-Sieg University of Applied Sciences, 53359 Rheinbach, Germany; 2 Department of Chemistry and Biology, University of Siegen, 57076 Siegen, Germany; 3 Department of Analytical Chemistry and Reference Materials, Federal Institute For Materials Research and Testing, 12200 Berlin, Germany

**Keywords:** Multivariate evaluation method, Pest infestations, Volatile organic compounds, Principal component analysis, Linear discriminant analysis, Plant sciences, Plant immunity, Plant signalling, Plant stress responses, Natural products, Biomarkers, Diagnostic markers, Analytical chemistry, Chemistry, Environmental chemistry, Environmental monitoring

## Abstract

Volatile organic compounds (VOCs) play an important role in the defense against pest infestations on plants. The analysis of these VOCs using gas chromatography mass spectrometry (GC-MS) enables the detection of pests by analyzing the VOC composition (VOC profiles) for specific patterns and markers. The analysis of such complex datasets with high biovariability poses a particular challenge. For this reason, a multivariate evaluation method based on a self-written Python script, using principal component analysis (PCA) and linear discriminant analysis (LDA), was developed and tested for functionality using a dataset, which has been evaluated manually and has identified five specific markers (2,4-dimethyl-1-heptene, 3-carene, $\alpha$-longipinene, cyclosativene, and copaene) for *Anoplophora glabripennis* (ALB) infestation on *Acer* trees. The results obtained in the present study did not only match the manually evaluated results, but lead to further insight into the dataset. Another sesquiterpene which is assumed to be $\alpha$-zingiberene was identified as an ALB specific marker in addition to 2,4-dimethyl-1-heptene and 3-carene. Furthermore, the European native beetle species goat moth *Cossus cossus* (CC) and poplar long-horned beetle *Saperda carcharias* (SC) were also analyzed for their VOCs to differentiate ALB specific VOC from other pest infestations. This comparison lead to the conclusion that the compounds $\alpha$-longipinene, cyclosativene, and copaene are not specific for ALB but for pest infestation in general. It was possible to identify not only specifically produced VOCs, but also differences in concentrations that arise specifically during ALB infestation. Therefore, the evaluation method for the detection of plant pests presented in this study represents a time-saving alternative to conventional non computing methods, which in addition provides more detailed results.

## Introduction

The emission of volatile organic compounds (VOCs) from plants as a defense mechanism against biotic and abiotic stress, and as a tool of communication with their environment, was first documented by Baldwin and Schulz^1^ as well as Rhoades^2^ in 1983. One of the most prominent examples is the attraction of insects for pollination, which is crucial for plant distribution and reproduction^3,4^. Additionally, VOCs play a defensive role; for instance, they are produced in response to pest and/or pathogen infestation^5,6^ or are emitted to attract natural predators of pests^4,6,7^. VOCs also serve as signals to neighboring plants, warning them of potential threats and enabling the early production of defensive VOCs in response^4–6^.

Because of these interactions the analysis of VOCs in plants can provide indications of possible pest and/or pathogen infestation. This has the advantage of early detection and therefore contributes to a rapid control and containment of the infestation^5,8^. Since VOCs are typically emitted as complex mixtures rather than as individual compounds, it is crucial to employ appropriate sampling and analytical methods to accurately assess the plants’ VOC profiles^9^.

In recent years, some progress has been made in the development of analysis methods using gas chromatography mass spectrometry (GC-MS)^10,11^, gas chromatography ion mobility spectrometry (GC-IMS)^11^, electronic nose^10,11^ and proton transfer reaction mass spectrometry (PTR-MS)^12^. The analysis of statically or dynamically enriched VOC samples using GC-MS has been established as one of the standard methods in all possible fields of expertise, e.g. disease detection^13–15^, environmental pollution^16,17^, food science^11,17,18^, and pharmacy^17,19^. However, the evaluation of VOC profiles obtained by GC-MS is still a major challenge^20–22^. In order to be able to detect possible pest and/or pathogen infestations in plants using VOC analysis, the VOC profiles of the plants must be examined for possible patterns and markers that specifically represent the pest or pathogen, while simultaneously discrimination against all background emissions can be achieved^23^. Due to the complexity and the many influencing factors that affect the VOC emission of plants, identifying such patterns manually is both very laborious and time-consuming^24^. A time-saving and facilitating evaluation process for such complex data is therefore of great advantage. Recent scientific studies are increasingly applying chemometric methods for the evaluation of chromatographic data, such as principal component analysis (PCA)^25,26^, partial least squares (PLS)^25,26^, or linear discriminant analysis (LDA)^25^. Software such as AMDIS^27^, PARADISe^28^, and OpenChrom^29^ are already available to facilitate the comparison and evaluation of data. However, there is still need and demand for improvements in the evaluation of such complex datasets in order to ensure an efficient and reliable detection method for pest infestations.

Therefore, this work features the development of an evaluation method to analyze GC-MS data of VOC measurements using chemometric methods in order to classify the data based on different VOC patterns and markers. For this purpose, a Python script was written that combines data merging, data preprocessing and chemometric evaluation for classification, which can be flexibly adapted to any dataset. In order to test the functionality of this evaluation procedure, a dataset that has already been evaluated manually is analyzed. The dataset is taken from the publications by Makarow et al., who have identified specific patterns and markers for an infestation of the invasive Asian longhorned beetle *Anoplophora glabripennis* (ALB) on maple trees (*Acer* L.)^30,31^. They had a statistically valid dataset of infested trees that represent biological variance. At the same time, the identified VOCs of interest occurred in 95–99% of all measurements^30^. In addition to the data of ALB those of the European native beetle species goat moth *Cossus cossus* (CC) and poplar long-horned beetle *Saperda carcharias* (SC) are also included in the analysis in order to exclude falsification of the pattern by native species. The data of CC and SC was obtained similarly to the data of ALB.

## Methods

The dataset from Makarow et al. includes samples from healthy trees (HT), ALB infested trees, SC infested trees/ larvae, and CC larvae^30^. In their work adsorption tubes filled with Tenax TA from Alltech Associates Inc. were used for sampling. All samples were taken with a flow rate of 30 mL/min for a duration of 90 min. Table 1 shows an overview of the analyzed samples and the used sampling procedure. The chromatographic analysis was performed using a 7890A/5975C inert XN MSD GC/MS device from Agilent Technologies coupled to a thermal desorption unit from Gerstel. GC was equipped with a DB5-MS capillary column from J&W (30m$\times$0.250 mm; 0.25 $\upmu$m).The mass spectra were recorded in the electron-impact mode (70 eV) from 30 to 400 DA. All other parameters regarding sampling procedure and the analytical methods can be found in the respective publications^30,31^.

Each chromatographic peak was annotated based on mass spectral comparison using the National Institute of Standards and Technology (NIST) 20 database. Diagnostic fragment ions (m/z) and literature Kovats retention indices (RI) were used to support compound identity, where available. Due to the retrospective nature of the data and changes in instrumentation, RI calibration using alkane standards was not feasible. Therefore, all compound names should be regarded as tentative. Key spectral data and match scores are provided in Supplementary Table S1.

**Table 1:** Overview of the analyzed samples, number of replicates, sampling method by Makarow et al.^30,31^ and the sampling description.

Sample	No. measurements	Sampling method	Description sampling method (Makarow et al.^30,31^)
HT	29	On the trunk	Trunk was wrapped in Nalophan foil Staples and tension belts were used for closing the foil Self-built adapter was used to adapt adsorbent tubes on the foil Pump was used to trap VOCs from the trunk on adsorbent tubes
ALB	16	On the trunk	See description HT
SC	17	Headspace-vial; on the trunk	Larvae were put into a 20 mL headspace vial Adsorbent tubes were put through the septum of the vial Pump was used to trap VOCs from larvae on the adsorbent tubes See description HT
CC	12	Headspace-vial	See description SC

In order to extract all possible markers for infestations the total ion chromatograms (TICs) of the data are used for evaluation. All TICs obtained from the respective data are converted into CSV-files using the OpenChrom Lablicate Edition 1.5.0 software and then analyzed using the custom-written script using Python 3.10, which consists of data selection, data preprocessing, and chemometric evaluation and will be explained in the following paragraph. The following packages and functions are used: *pandas* for handling data, *os* for interaction with the operating system, *datetime* for timestamps, *NumPy* for mathematical operations, *SciPy* for signal processing, *Matplotlib pyplot* and *plotly* for visualization, *seaborn* for heatmaps, and *Sci-Kit learn* for chemometric analysis.

In the data selection process, folders are created for test and train data containing all CSV files of the classes to be analyzed (HT, ALB, CC and SC). Due to the availability of data and the time-consuming data acquisition, it is not possible to work with a large amount of data, despite the fact that it is not an ideal condition for a chemometric evaluation. For each class, 10 data files are chosen randomly as train data, all other data files are declared as test data. The script extracts both retention time and TIC for each measurement. Because the first 3 min and the last 4 min of the chromatograms only show impurities, these data areas are cut off. Subsequently, the extracted data undergo preprocessing, where the datasets are smoothed, the baseline is corrected, and the chromatograms are normalized to their total area. A Savitzky-Golay filter with polynomial order 3 and width 11 is used for smoothing. The baseline correction is performed using the Asymmetric Least Squares Smoothing method with a smoothing factor of $10^6$, an asymmetry parameter of 0.001 and a number of 10 iterations. The resulting baseline is then subtracted from the respective chromatograms. Afterwards, total area normalization is performed on the chromatograms in order to obtain comparable intensities without changing the height ratio of the signals and thus enable a qualitative determination of compounds. Lastly, a signal threshold of 3 % of the normalized intensity of each chromatogram is chosen to focus on peaks and to minimize background noise. An exemplary chromatogram before and after preprocessing can be found in the Supplementary figure S1. The preprocessed data are then merged into a single train or test dataset, respectively.

**Fig. 1:**
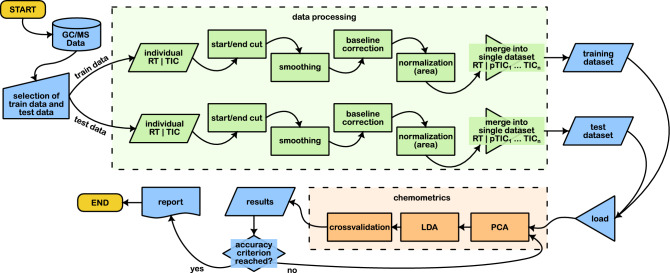
Flowchart of the Python script. The relevant steps of data selction, data processing, and chemometrical evaluation are shown. GC/MS: gas chromatography mass spectrometry, RT: retention time, TIC: total ion chromatogramm, PCA: principal component analysis, LDA: linear discriminant analysis.

The preprocessed train and test datasets are then analyzed chemometrically. For this purpose, data reduction is performed on the train dataset using principal component analysis (PCA). The optimum number of PCs used for further analysis is chosen manually and is determined based on the accuracy of the train and test data. PCs are added until the accuracy of the test data deteriorates, indicating that overfitting has occurred^32,33^. Hence, the the optimum number of PCs is the number before overfitting arises. The dimensionally reduced train dataset is subsequently classified using linear discriminant analysis (LDA). To check the suitability of the LDA, a “k-fold” crossvalidation (k-fold-CV) with 10 iterations as well as a “leave one out” crossvalidation (loo-CV) is performed on the train data. The k-fold-CV is normally used for large amounts of data, while the loo-CV is favored for small amounts of data. Next, the test dataset is dimensionally reduced with the calculated PCA function of the train dataset and classified with the calculated discriminant function of the LDA. Finally, the accuracy of the classified test data is checked using the *SciKit-learn* package. The script’s workflow is shown in Fig. 1.

## Results and discussion

**Fig. 2:**
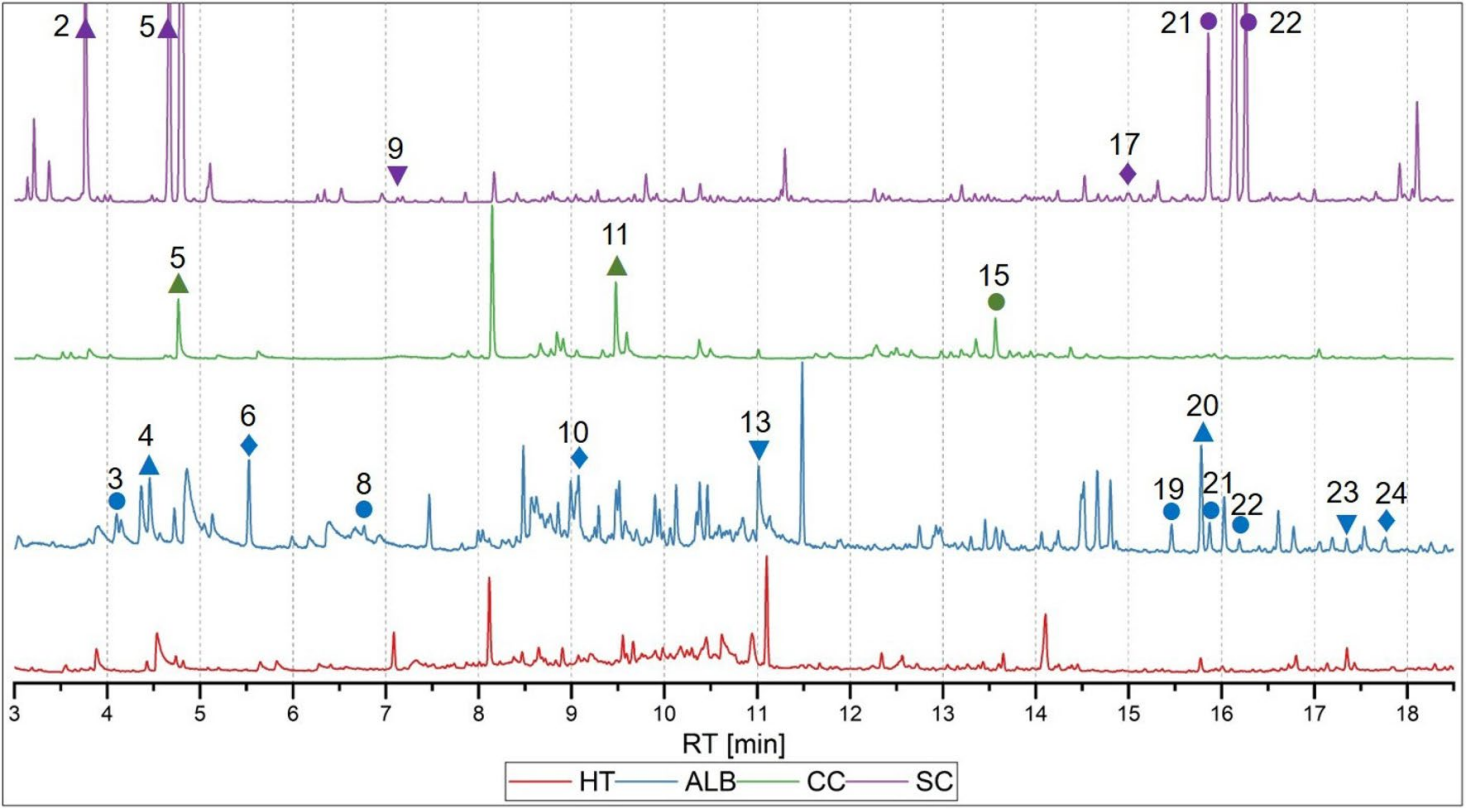
Stacking of GC-MS example chromatograms of the four investigated classes. Red: healthy *Acer* tree (HT), blue: *Anoplophora glabripennis* (ALB) infested *Acer* tree, green: *Cossus cossus* (CC) larvae, purple: *Saperda carcharias* (SC) infested *Acer* tree; RT: retention time. Compounds specific to one pest are marked with diamond symbol. Compounds which occur during pest infestation are labeled with cycle symbol. Increasing concentrations of compounds during infestation are characterized with an upward triangle symbol, decreasing concentrations with a downward triangle symbol, respectively. The peak No. can be assigned to the compounds in Table 3.

**Fig. 3:**
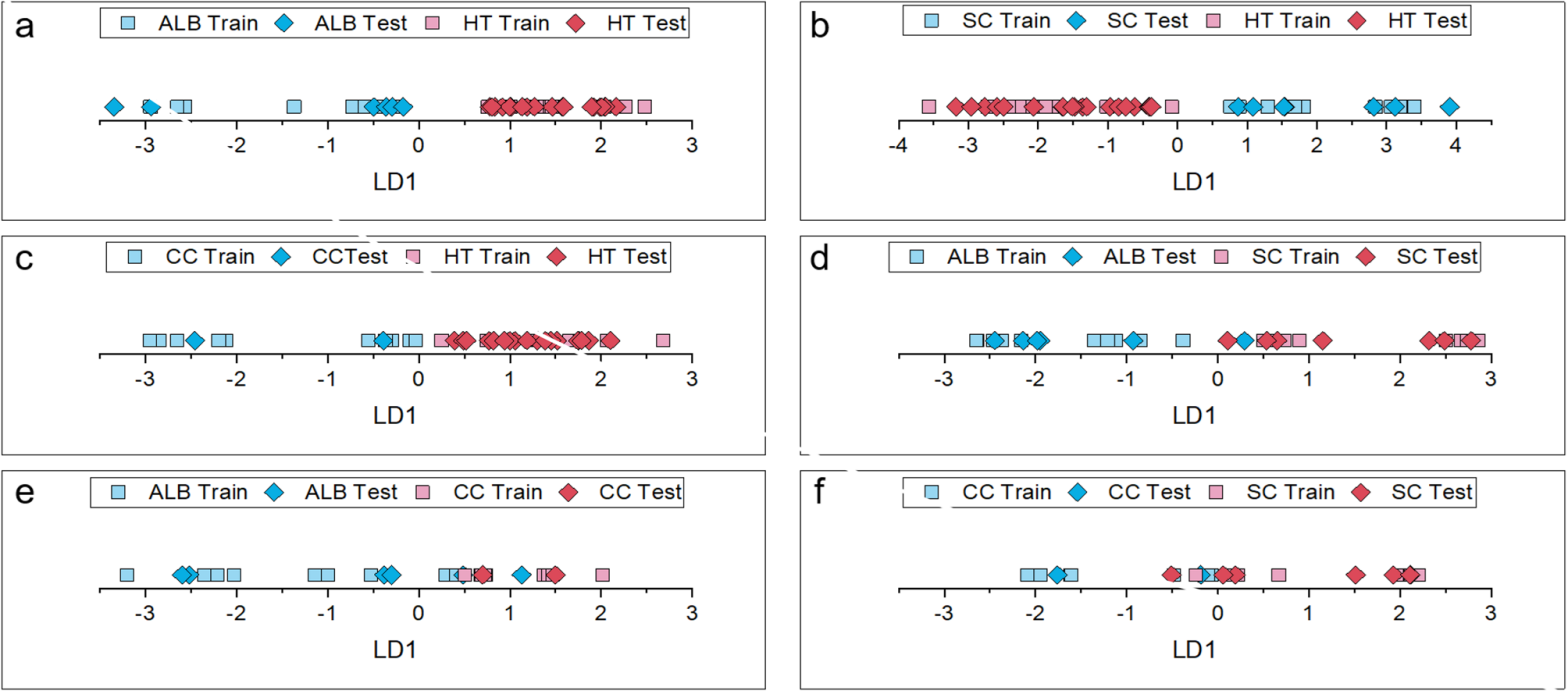
LD-Plots of the six different class comparisons. **(a)** *Anoplophora glabripennis* (ALB) infested *Acer* tree and healthy tree (HT), **(b)** *Saperda carcharias* (SC) larvae infested *Acer* tree and HT, **(c)** *Cossus cossus* (CC) larvae and HT, **(d)** ALB infested *Acer* tree and SC larvae infested *Acer* tree, **(e)** ALB infested *Acer* tree and CC larvae, **(f)** SC larvae infested *Acer* tree and CC larvae.

In order to detect specific VOC-markers for ALB infestation using GC-MS, data from healthy trees (HT), ALB infested *Acer* trees, SC larvae and infested *Acer* trees, and CC larvae were used. Figure 2 shows an exemplary and representative chromatogram for each class. Six class comparisons were created from the four classes: ALB - HT, SC - HT, CC - HT, ALB - SC, ALB - CC and SC - CC. The most important parameters for the chemometric evaluation are listed in Table 2. All six class comparisons could be adequately classified with the dimensionally reduced datasets (Fig. 3).

**Fig. 4:**
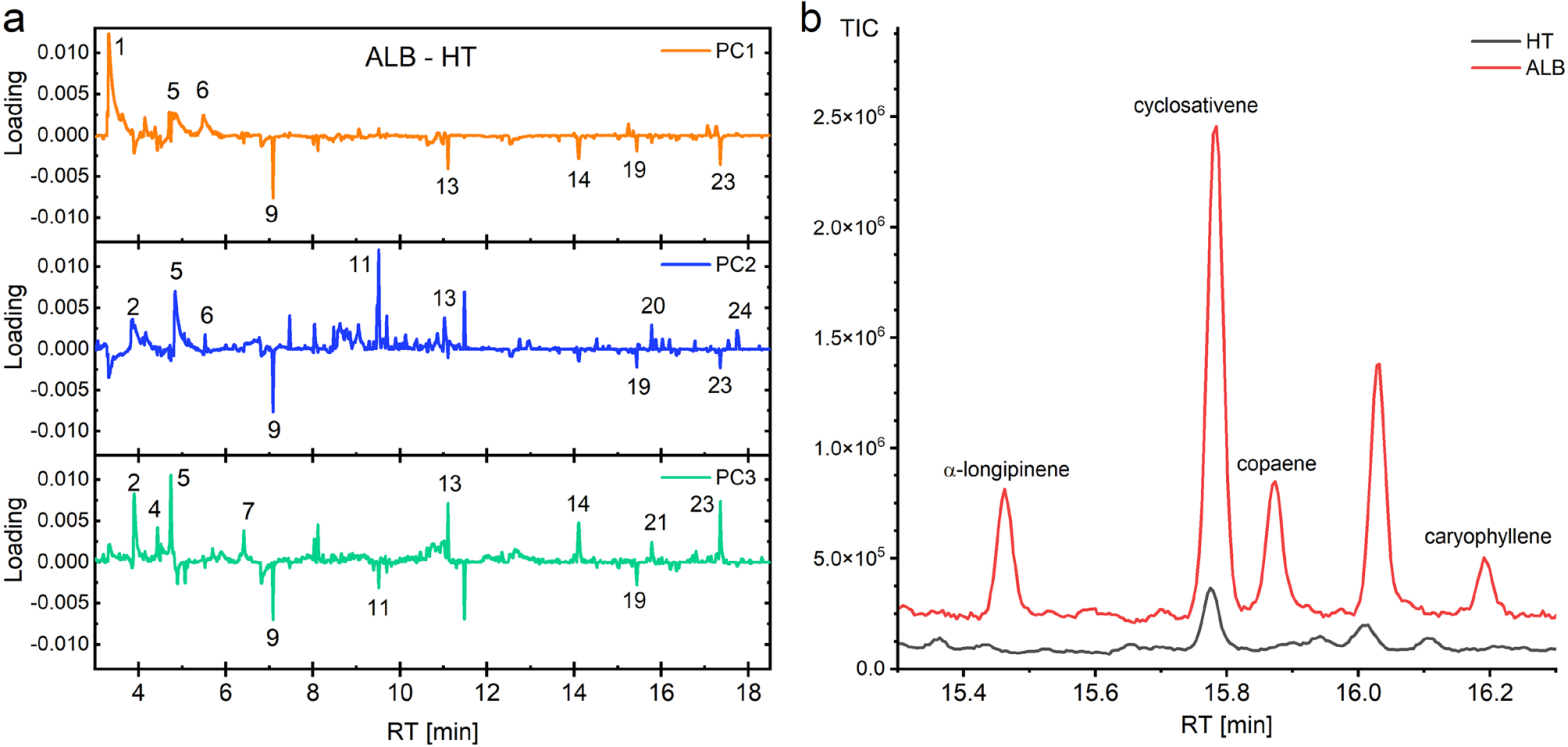
**(a)** Loading plots of the class comparison of *Anoplophora glabripennis* (ALB) infested *Acer* tree and healthy tree (HT). PC: principal component, RT: retention time, the Peak No. can be assigned to the compounds in Table 3; **(b)** overlay of example chromatograms of HT and ALB between retention time 15.2 min and 16.3 min with identified compounds cyclosativene, $\alpha$-longipinene, copaene, and caryophyllene. Red: ALB, black: HT.

The results of the crossvalidations and the accuracy of the test datasets as well as the number and variance of the principal components (PCs) are listed in Table 2. Based on the loadings of the dimensionally reduced data, it is possible to understand which retention times and thus which compounds have the greatest influence on the classification. Figure 4a exemplarily shows the loading plots of the PCA, where only the data of ALB vs. HT were investigated. The loading plots of all other comparisons, a 2D and 3D LD plot as well as all score plots can be found in the Supplementary Figures S2–S14. Table 3 shows all important retention times found in the loadings and their corresponding compounds determined through comparison with the NIST 20 database. A total of 24 compounds (10 hydrocarbons (HC), 2 aromatic hydrocarbon (AHC), 3 monoterpenes (MT), 5 sesquiterpenes (ST) and 4 unknown compounds) can be considered as potential markers for pests on trees and are displayed in the following subsections. Although Kovats’ retention indices (RI) for compound 6 and 24 are slightly lower than those for compound 5 and 23, their spectral signatures and retention behavior suggest they are presumably styrene and presumably $\alpha$-zingiberene, respectively. Both annotations are based on strong spectral similarity (NIST match >90%) and consistent elution behavior relative to known RI values on DB-5 columns. Three additional compounds from the class of cyclosiloxanes can be excluded as potential markers, as they are typical contaminants in gas chromatography when PDMS-based stationary phases are used (e.g. due to column bleeding)^34^ and also are emitted by a large number of personal care products and thus occur due to contamination^35^.

**Table 2:** Overview of the chemometric parameters for the analyzed class comparisons.

Class comparison	No. of PCs	Variance of PCs, %	CV-score, %	Accuracy, %
ALB - HT	3	PC1: 21.32 PC2: 19.45 PC3: 12.29 Total: 53.06	k-fold: 100 loo: 100	ALB: 100 HT: 100
SC - HT	3	PC1: 24.14 PC2: 16.25 PC3: 12.78 Total: 53.17	k-fold: 95 loo: 100	SC: 100 HT: 100
CC - HT	3	PC1: 84.50 PC2: 4.43 PC3: 3.17 Total: 92.10	k-fold: 85 loo: 100	CC: 100 HT: 100
ALB - SC	6	PC1: 23.41 PC2: 20.97 PC3: 12.38 PC4: 8.98 PC5: 7.95 PC6: 5.52 Total: 79.21	k-fold: 100 loo: 100	ALB: 83 SC: 100
ALB - CC	4	PC1: 79.24 PC2: 5.86 PC3: 4.65 PC4: 2.65 Total: 92.40	k-fold: 80 loo: 85	ALB: 67 CC: 100
SC - CC	4	PC1: 89.58 PC2: 3.40 PC3: 2.30 PC4: 1.58 Total: 96.58	k-fold: 80 loo: 85	SC: 86 CC: 100

**Table 3:** List of possible marker compounds for the different class comparisons determined via chemometric analysis, including a categorization of the loading values. All compound names are tentative assignments based on NIST 20 spectral comparison and literature RI values. For diagnostic fragment ions and match scores, see Supplementary Table S1. RT: Retention time; AHC: aromatic hydrocarbons, HC: hydrocarbons, MT: monoterpenes, ST: sesquiterpenes; RI: Kovats retention index for non polar columns according to the pherobase database, ALB: *Anoplophora glabripennis*, HT: Healthy tree, SC: *Saperda carcharias*, CC: *Cossus cossus*. Loading values (LV): LV $<{0.005}:~\vartriangle$, LV $<{0.010}:~\vartriangle \vartriangle$, LV $\ge {0.010}:~\vartriangle \vartriangle \vartriangle$, LV $>{-0.005}:~\triangledown$, LV $>{-0.010}:~\triangledown \triangledown$, LV $\le {-0.010}:~\triangledown \triangledown \triangledown$, –: no differences in comparison.

Peak no.	RT, min	Class	CAS-no.	Compound	RI	ALB-HT	SC-HT	CC-HT	ALB-SC	ALB-CC	SC-CC
1	3.3	–	–	Unknown	–	$\vartriangle \vartriangle \vartriangle$/$\triangledown$	–	–	$\vartriangle \vartriangle \vartriangle$/$\triangledown \triangledown \triangledown$	$\vartriangle \vartriangle \vartriangle$	–
2	3.8	AHC	108-88-3	Toluene	762	$\vartriangle \vartriangle$/$\triangledown$	$\vartriangle \vartriangle$/$\triangledown$	$\vartriangle \vartriangle$/$\triangledown \triangledown$	$\vartriangle$/$\triangledown$	$\vartriangle$	$\vartriangle \vartriangle$/$\triangledown$
3	4.1	HC	589-53-7	4-Methyl heptane	770	$\vartriangle$	–	–	$\vartriangle$/$\triangledown$	$\vartriangle$	$\triangledown$
4	4.4	HC	111-65-9	*n*-Octane	800	$\vartriangle$/$\triangledown$	$\vartriangle$/$\triangledown$	$\vartriangle$/$\triangledown$	–	$\vartriangle$	–
5	4.7	HC	66-25-1	Hexanal	800	$\vartriangle \vartriangle \vartriangle$/$\triangledown$	$\vartriangle \vartriangle$/$\triangledown \triangledown \triangledown$	$\vartriangle \vartriangle$/$\triangledown \triangledown$	$\vartriangle \vartriangle$/$\triangledown \triangledown \triangledown$	$\vartriangle \vartriangle$/$\triangledown$	$\vartriangle \vartriangle$/$\triangledown$
6	5.5	HC	19549-87-2	2,4-Dimethyl- 1-heptene	830	$\vartriangle$	–	–	$\vartriangle$	$\vartriangle$/$\triangledown$	–
7	6.4	HC	111-84-2	*n*-Nonane	900	$\vartriangle$	$\vartriangle$	$\vartriangle$/$\triangledown$	$\vartriangle \vartriangle \vartriangle$	–	$\vartriangle \vartriangle \vartriangle$/$\triangledown \triangledown$
8	6.8	AHC	100-42-5	Styrene	893	$\triangledown$	$\vartriangle \vartriangle$	$\vartriangle$	–	–	–
9	7.1	MT	7785-70-8	$\alpha$-Pinene	934	$\triangledown \triangledown$	$\vartriangle \vartriangle \vartriangle$	$\vartriangle$ $\vartriangle \vartriangle$	$\vartriangle$	$\vartriangle$	–
10	9.1	MT	13466-78-9	3-Carene	1011	$\vartriangle$	–	–	$\vartriangle$	$\vartriangle$	–
11	9.5	MT	138-86-3	Limonene	1047	$\vartriangle \vartriangle \vartriangle$/$\triangledown$	–	$\triangledown \triangledown$	$\vartriangle \vartriangle$/$\triangledown \triangledown$	$\vartriangle \vartriangle \vartriangle$/$\triangledown \triangledown$	$\vartriangle \vartriangle \vartriangle$/$\triangledown$
12	10.4	HC	1120-21-4	Undecane	1100	–	–	$\triangledown$	–	$\vartriangle$	–
13	11.1	HC	124-19-6	Nonanal	1104	$\vartriangle \vartriangle$/$\triangledown$	$\vartriangle \vartriangle$/$\triangledown$	$\vartriangle \vartriangle$/$\triangledown$	$\vartriangle$/$\triangledown$	$\vartriangle \vartriangle$	$\triangledown$
14	12.5	HC	112-31-2	Decanal	1209	$\vartriangle$/$\triangledown$	$\triangledown$	–	–	–	–
15	13.5	–	–	Unkown	–	–	–	$\triangledown$	–	$\triangledown$	$\vartriangle$
16	14.1	HC	629-50-5	Tridecane	1300	$\vartriangle$/$\triangledown$	$\vartriangle \vartriangle$/$\triangledown$	$\vartriangle$/$\triangledown$	–	–	–
17	15.0	–	–	Unknown	–	–	$\vartriangle$	$\vartriangle$	–	–	–
18	15.2	–	–	Unknown	–	$\vartriangle$	–	–	–	$\vartriangle$	–
19	15.5	ST	5989-08-2	$\alpha$-Longipinene	1351	$\triangledown$	$\vartriangle \vartriangle$	$\vartriangle \vartriangle$	–	–	–
20	15.8	ST	22469-52-9	Cyclosativene	1368	$\vartriangle$	$\vartriangle$/$\triangledown$	$\vartriangle$/$\triangledown$	$\vartriangle$	$\vartriangle$	$\vartriangle$
21	15.9	ST	3856-25-5	Copaene	1376	$\vartriangle$	$\triangledown$	–	$\triangledown$	–	–
22	16.3	ST	87-44-5	Caryophyllene	1428	$\vartriangle$/$\triangledown$	$\triangledown$	–	$\vartriangle$/$\triangledown \triangledown$	$\vartriangle$	$\vartriangle \vartriangle$/$\triangledown$
23	17.4	HC	629-62-9	Pentadecane	1500	$\vartriangle \vartriangle$/$\triangledown$	$\vartriangle \vartriangle$/$\triangledown \triangledown$	$\vartriangle \vartriangle$/$\triangledown \triangledown$	$\vartriangle$	$\vartriangle$	–
24	17.7	ST	495-60-3	$\alpha$-Zingiberene	1495	$\vartriangle$	–	–	$\vartriangle$/$\triangledown$	$\vartriangle$	–

### Potential markers of the six class comparisons

21 compounds (7 HCs, 2 AHC, 3 MTs, 4 STs and 5 unknown compounds) were identified as differences between ALB and healthy tree (HT). Regarding the chromatograms of ALB and HT (Fig. 2), 8 compounds (4-methylheptane, 2,4-dimethyl-1-heptene, presumably styrene, 3-carene, $\alpha$-longipinene, copaene, caryophyllene and presumably $\alpha$-zingiberene) are only emitted during ALB infestation whereas 2 compounds ($\alpha$-pinene and decanal) occur exclusively in HT. *n*-octane, hexanal, *n*-nonane, limonene and cyclosativene increase in concentration by ALB infestation, while the emissions of nonanal, tridecane and pentadecane decrease.

For the distinction between SC and HT, 15 compounds (7 HCs, 2 AHC, 1 MTs, 4 STs and 1 unknown compounds were characterized. Regarding Fig. 2, copaene, caryophyllene and one unknown compound are exclusively emitted by SC infestation, whereas *n*-octane, presumably styrene, nonanal, decanal, tridecane, cyclosativene and pentadecane only occur in HT. The concentrations of toluene and hexanal increase by SC infestation, while the emission of $\alpha$-pinene decreases.

For the comparison of CC and HT, 15 compounds (7 HCs, 2 AHC, 2 MTs, 2 STs and 2 unknown compounds) could be distinguished. One unkown compound is exclusively emitted from CC, while *n*-octane, presumably styrene, $\alpha$-pinene, nonanal, cyclosativene and pentadecane are emitted only by HT. Undecane and toluene are higher concentrated in HT, whereas hexanal and limonene are more emitted by CC.

When comparing the native beetle species with the ALB, 16 compounds (8 HCs, 1 AHC, 3 MTs, 3 STs and 1 unknown compounds) are distinguished between ALB and SC. Regarding Fig. 2, 9 compounds (*n*-octane, 2,4-dimethyl-1-heptene, 3-carene, limonene, undecane, nonanal, cyclosativene, pentadecane and presumably $\alpha$-zingiberene) are emitted exclusively by ALB infested trees, whereas only one unknown compound is specific for SC infestation. *n*-nonane is more emitted by ALB infested trees while toluene, hexanal, copaene and caryophyllene are more emitted by SC infested tree respectively.

17 compounds (8 HCs, 1 AHC, 3 MTs, 2 STs and 3 unknown compounds) between ALB and CC are determined. None of the compounds are CC specific, but 9 compounds (*n*-octane, 2,4-dimethyl-1-heptene, 3-carene, nonanal, cyclosativene, copaene, caryophyllene, pentadecane and presumably $\alpha$-zingiberene) are emitted only upon ALB infestation. The compounds hexanal, limonene, and 1 unkown compound are higher concentrated in CC, while toluene, $\alpha$-pinene, and undecane are more emitted by ALB infested trees.

9 compounds (5 HCs, 1 AHC, 1 MT, and 2 STs) could be determined as differences for the comparison between SC and CC. limonene, nonanal, and 1 unknown compound are CC specific, while *n*-nonane, cyclosativene, and caryophyllene are emitted only by SC infestation. In addition, the compounds toluene and hexanal are higher concentrated in SC.

### ALB specific markers

Makarow et al. found that ALB larvae, ovipositions, and imagos emit a combination of the compounds copaene, cyclosativene, and 2,4-dimethyl-1-heptene^31^. When examining ALB-infected trees, they also found the compounds $\alpha$-longipinene and 3-carene. Furthermore, caryophyllene could only be detected in trees infected with ALB and not in trees exposed to mechanically induced stress^30^. These results could be partially confirmed and extended in the chemometric data analysis applied in this study.

Regarding the 24 compounds identified as potential markers via chemometric analysis and the results obtained from comparison of the different classes a total of 14 potential ALB-specific compounds were identified in the analysis of the ALB-HT, ALB-SC and ALB-CC class comparisons. However, only 2,4-dimethyl-1-heptene, 3-carene, and presumably $\alpha$-zingiberene could be identified as actually ALB-specific in the analysis of class comparisons, while 4-methylheptane, presumably styrene, $\alpha$-longipinene, copaene, and caryophyllene are also emitted by SC-infested trees as well as the CC. Therefore, the results of Makarow et al.^30^ regarding 2,4-dimethyl-1-heptene and 3-carene as specific to ALB infestation can be confirmed, but for the compounds copaene, cyclosativene, caryophyllene, and $\alpha$-longipinene (Fig. 4b) it is reasonable to assume that these compounds are not ALB-specific compounds, but rather compounds that are produced in greater quantities upon induction of biotic stress. This assumption can be verified by consulting the literature and will be discussed in detail in the following paragraph^36,37^.

Rodriguez-Saona et al. found that gypsy moth infestation on the shrub *Vaccinium corymbosum* induces a high emission of caryophyllene^38^. Slavik et al. observed that infection with the fungi *Botrytis cinerea* and *Oidium neolycopesici* leads to emission of copaene in tomato plants^39^. Tomato plants also emit caryophyllene when infected with *Bemisia tabaci*^40^. It was established that an increased concentration of caryophyllene increases the resistance of plants to pests^40,41^. Furthermore, Li et al. discovered that increased concentrations of cyclosativene, $\alpha$-longipinene and caryophyllene are observed in borings of the Japanese pine sawyer *Monochamus alernatus* on *Pinus massoniana*^42^. The increased production of copaene, caryophyllene, $\alpha$-longipinene and cyclosativene during herbivore infestation is due to a defense mechanism of the plants by activation of the jasmonic acid pathway^43,44^. Erb et al. induced roots and leaves of maize plants with jasmonic acid and observed a strong increase in the concentration of copaene, caryophyllene and cyclosativene^36^. During *Spodoptera litoralis* infestation on *Medicago truncatula*, Leitner et al. determined both a local increase in jasmonic acid concentration and a strong increase in the emission of caryophyllene, copaene, and cyclosativene^45^.

In addition to the results of Makarow et al.^30,31^, presumably $\alpha$-zingiberene was identified as a specific marker for ALB infestation using the evaluation method described in this study. A literature search revealed that $\alpha$-zingiberene could also be detected in maize infested with *Spodoptera frugiperda* females^46^. No publication was found that identifies $\alpha$-zingiberene in association with an ALB infestation.

However, the evaluation method applied in this study not only identified specifically produced VOCs, but also differences in concentrations that arise specifically during ALB infestation. Thus, the compound *n*-octane shows a higher concentration during ALB infestation, while the emission of nonanal and pentadecane is reduced. *n*-octane was found by Laothawornkitkul et al. in tomato leaves^47^. Pentadecane serves, besides other roles, as a messenger compound for pollination in plants^48^, while the emission of nonanal can attract predator insects upon herbivore infestation^49,50^. The fact that both compounds are emitted in lower concentrations during ALB infestation suggests that either the tree is no longer able to produce these compounds sufficiently due to the infestation or that the ALB actively inhibits the emission in order to prevent the attraction of possible predators.

### SC and CC specific markers

In addition to ALB, the two non-invasive herbivores SC and CC were examined for specific markers in this publication. For SC, three potentially specific compounds could be identified: copaene, caryophyllene and an unknown compound. However, copaene and caryophyllene are compounds that are generally emitted during herbivore infestation and are therefore not specific to a particular pest^38–41^, leaving the unknown compound to be the only one specific to SC-infested trees. Nevertheless, changes in the concentration of certain compounds are also an indication of SC infestation. Thus, an increased concentration of toluene and hexanal, as well as a reduced emission of $\alpha$-pinene was found in SC infestations. Sriprapat et al. were able to prove that plants absorb the pollutant toluene from the ambient air^51^. The differences in concentration can therefore not be assigned to the plant or the infestation with SC, but are rather a result of polluted ambient air. Hexanal is an inhibitor of phospholipase D (PLD), which is among other functions responsible for the maintenance of cell physiology and cell membrane hydrolysis^52^. Inhibitors cause, among other effects, proliferation, meaning rapid cell growth^53^. An increased concentration of hexanal therefore indicates a defense mechanism of the plant, which tries to counteract the damage caused by the SC through increased cell growth. Ortiz-Carreon et al. also found a reduced concentration of $\alpha$-pinene in maize plants infested with *Spodoptera frugiperda*^51^.

No specific markers could be identified for CC. One unkown compound could be found in CC, but not in HT. However, small amounts of this unknown compound could also be detected in ALB infestation. Furthermore, higher concentrations of hexanal and limonene were detected in CC compared to HT and could be an indication of CC infestation.

For completeness, 4-methylheptane and presumably styrene, which were emitted by all three herbivore infestations, but not by HT, will be discussed. Literature research on these two compounds revealed that styrene is a very common VOCs in the plant environment^54,55^ and was reported to have inhibitory properties on bacterial^56^ and fungal infections^57^. For 4-methyl heptane no literature on herbivore infestation could be found. Literature research only revealed that it was detected in raw amaranth seeds^58^.

## Conclusion

This study introduces a robust multivariate evaluation method for analyzing VOCs to detect pest infestations in plants. By integrating data merging, preprocessing, and advanced chemometric techniques, the approach offers a more efficient and objective alternative to traditional manual evaluation of GC-MS data. The method confirms and refines previous findings by Makarow et al.^30,31^, with 2,4-dimethyl-1-heptene and 3-carene reaffirmed as reliable markers for ALB infestation. Additionally, another sequiterpene, which is assumed to be $\alpha$-zingiberene was identified as a novel, ALB-specific marker, and concentration changes in nonanal and pentadecane were observed in infested samples. The method also enabled the identification of VOC markers associated with other pest species such as SC and CC, demonstrating its ability to distinguish between different types of infestations.

A key strength of this evaluation method lies in its adaptability and speed. It can be easily applied to various plant health questions and GC-MS setups with minimal adjustment. This facilitates the identification of disease- or pest-specific VOC patterns more quickly, which is particularly beneficial for both laboratory-based studies and potential field applications. In conclusion, the presented approach offers a solid and scalable framework for VOC-based pest and pathogen detection, contributing to more effective plant monitoring strategies and advancing the use of chemometric tools in agricultural and ecological research.

## Supplementary Information

Supplementary Information.

## Data Availability

The authors declare that the data is provided within the manuscript and its Supplementary Information files. Should any raw data files be needed in another format they are available from the corresponding author upon reasonable request. The Python script is available at GitHub.com: “https://github.com/Sarahvrmrn/Multivariate-evaluation-method-/blob/main/ALB_Evaluation.py”.
