# Supplementary material for: Multivariate evaluation method for the detection of pest infestations on plants via VOC analysis using gas chromatography mass spectrometry
Source: Sci Rep. 2025 Jul 16;15:25858. doi: 10.1038/s41598-025-11607-5 (PMC12267415; doi:10.1038/s41598-025-11607-5)
Supplement: Supplementary file 1 — Supplementary Information. [file 41598_2025_11607_MOESM1_ESM.pdf]

Supplementary Information

Table S1: Overview of peak identification using NIST comparison and Kovats RIs.

Table 1: Overview of chromatographically relevant compounds. For each compound, the peak number, retention time (RT), CAS number, compound name, characteristic mass fragments (m/z), Kovats retention index (RI) for nonpolar columns according to the pherobase database, and the match score from NIST 20 are listed. Due to the lack of RI calibration and standard validation, all compound assignments are tentative.

Peak No.	RT, min	CAS-No.	Compound	Major fragments (m/z)	RI	NIST Match, %
1	3.3	---	Unknown	---	---	---
2	3.8	108-88-3	toluene	39, 43, 51, 65, 91	762	94
3	4.1	589-53-7	4-methyl heptane	43, 55, 70, 114	770	90
4	4.4	111-65-9	*n*-octane	43, 57, 71, 85, 114	800	93
5	4.7	66-25-1	hexanal	44, 56, 72, 82	800	97
6	5.5	19549-87-2	2,4-dimethyl-1-heptene	43, 55, 70, 83, 126	830	94
7	6.4	111-84-2	*n*-nonane	43, 57, 71, 85, 99, 128	900	96
8	6.8	100-42-5	styrene	51, 63, 78, 104	893	93
9	7.1	7785-70-8	α-pinene	40, 55, 67, 77, 93, 105, 121, 136	934	97
10	9.1	13466-78-9	3-carene	41, 57, 67, 79, 93, 105, 121, 136	1011	92
11	9.5	138-86-3	limonene	40, 53, 68, 79, 93, 107, 121, 136	1047	99
12	10.4	1120-21-4	undecane	43, 57, 71, 85, 98, 113, 127, 156	1100	90
13	11.1	124-19-6	nonanal	41, 57, 70, 82, 98, 114	1104	96
14	12.5	112-31-2	decanal	43, 57, 0, 82, 95, 112, 128	1209	91
15	13.5	---	Unknown	---	---	---
16	14.1	629-50-5	tridecane	43, 57, 71, 85, 99, 112, 127, 141, 155, 184	1300	90
17	15.0	---	Unknown	---	---	---
18	15.2	---	Unknown	---	---	---
19	15.5	5989-08-2	α-longipinene	40, 55, 69, 77, 93, 105, 119, 133, 147, 161, 189, 204	1351	98
20	15.8	22469-52-9	cyclosativene	40, 55, 79, 94, 105, 119, 133, 147, 161, 175, 189, 204	1368	99
21	15.9	3856-25-5	copaene	40, 55, 69, 93, 105, 119, 161, 204	1376	95
22	16.3	87-44-5	caryophyllene	40, 55, 69, 79, 93, 105, 120, 133, 147, 161, 175, 189, 204	1428	99
23	17.4	629-62-9	pentadecane	43, 57, 71, 85, 99, 113, 127, 141, 155, 169, 193, 212	1500	97
24	17.7	495-60-3	α-zingiberene	41, 56, 69, 77, 93, 99, 105, 204	1495	91

Figure S1: Comparison of an exemplary chromatogram of an *Anoplophora glabripennis* (ALB) infested *Acer* tree before and after preprocessing

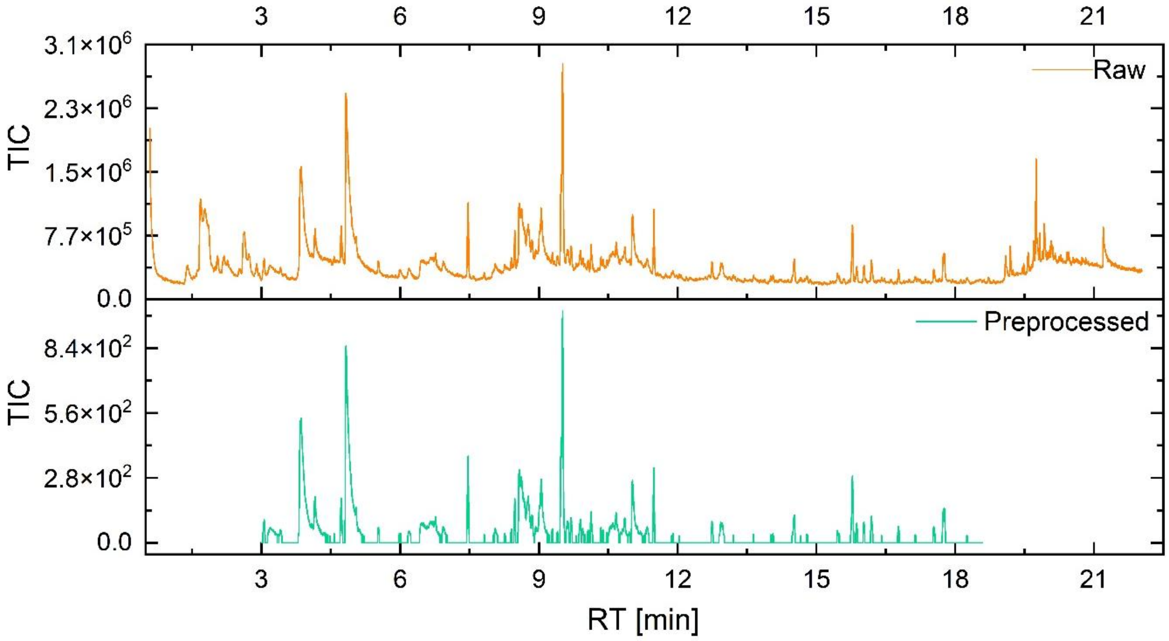

Figure S1: Exemplary chromatogram of an ALB infested *Acer* tree before and after preprocessing: Orange: raw data, green: preprocessed data.

Figure S2: Principal Component Analysis (PCA) - Loading plots of the class comparison poplar long-horned beetle *Saperda carcharias* (SC) vs. Healthy tree (HT)

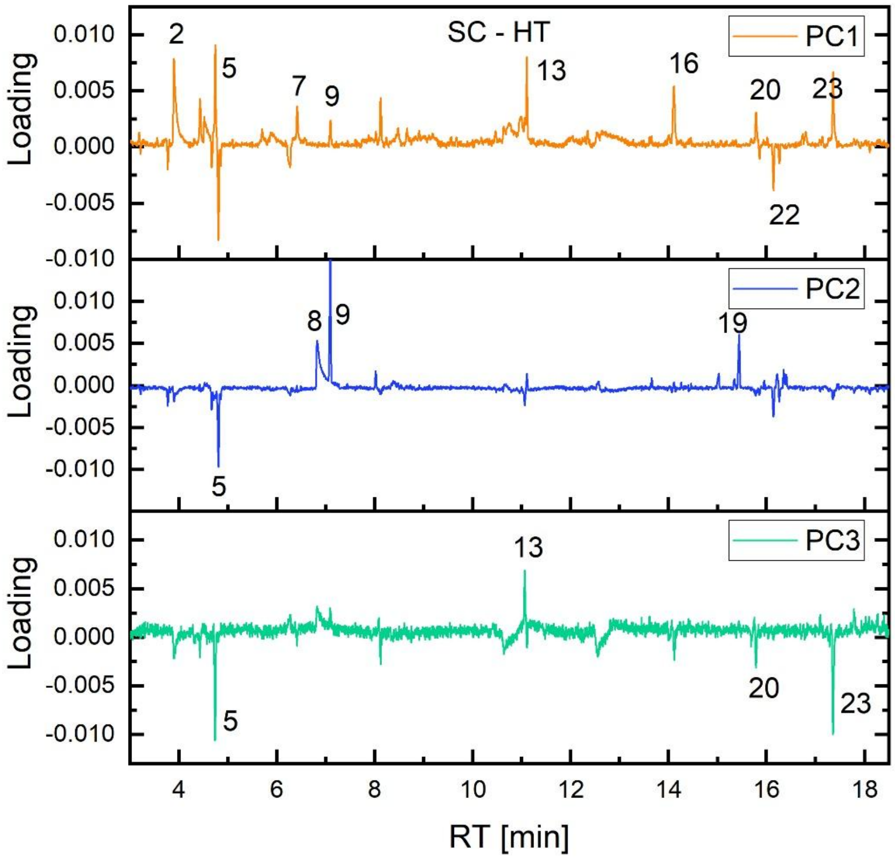

Figure S2. PCA - Loading plots of SC - HT: Orange: PC1, blue: PC2, green: PC3.

Figure S3: Principal Component Analysis (PCA) - Loading plots of the class comparison goat moth *Cossus cossus* (CC) vs. Healthy tree (HT)

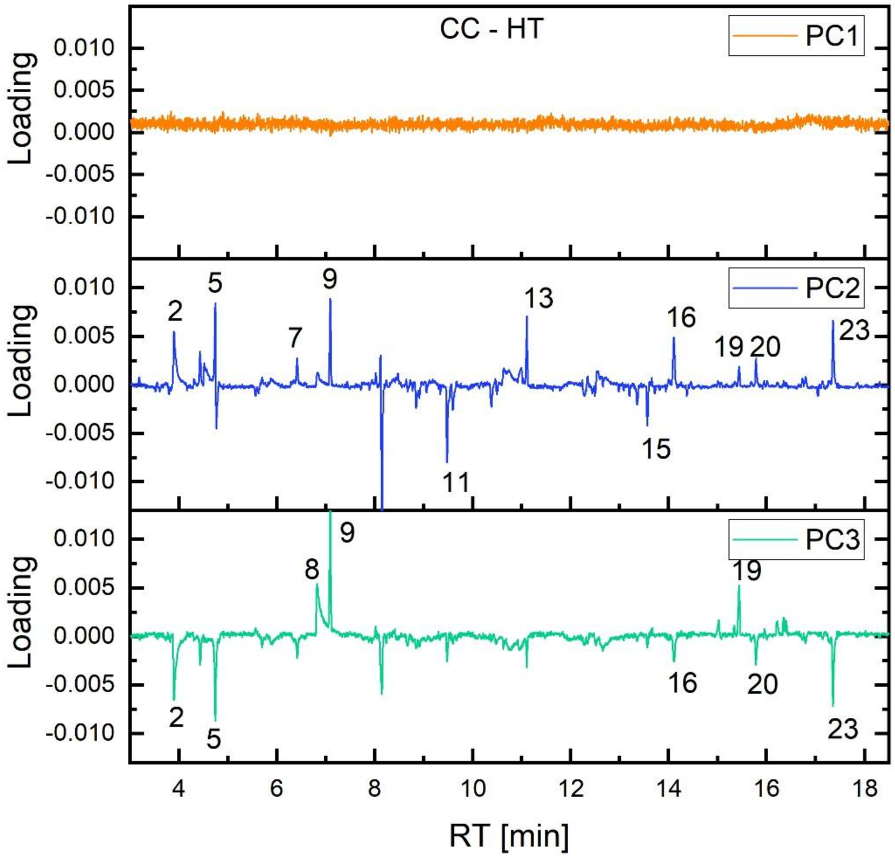

Figure S3. PCA - Loading plots of CC - HT: Orange: PC1, blue: PC2, green: PC3. The noise of PC1 is due to the fact, that for CC only data of larvae and not infested tree were available. CC larvae emit less VOC than trees in general, so the signal to noise ratio of CC data is higher than in the other classes after normalization.

Figure S4: Principal Component Analysis (PCA) - Loading plots of the class comparison Asian longhorned beetle *Anoplophora glabripennis* (ALB) vs. poplar long-horned beetle *Saperda carcharias* (SC)

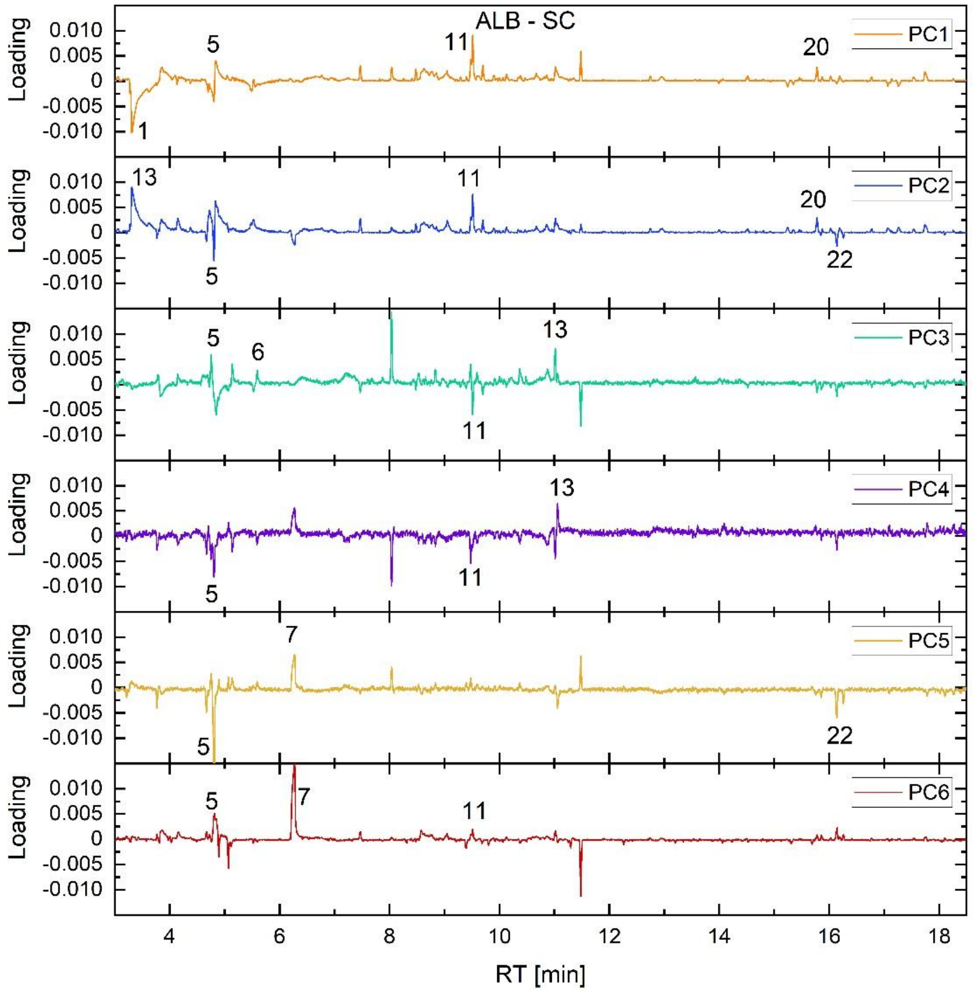

Figure S4. PCA - Loading plots of ALB - SC: Orange: PC1, blue: PC2, green. PC3, purple: PC4, yellow: PC5, red: PC6.

Figure S5: Principal Component Analysis (PCA) - Loading plots of the class comparison Asian longhorned beetle *Anoplophora glabripennis* (ALB) vs. goat moth *Cossus cossus* (CC)

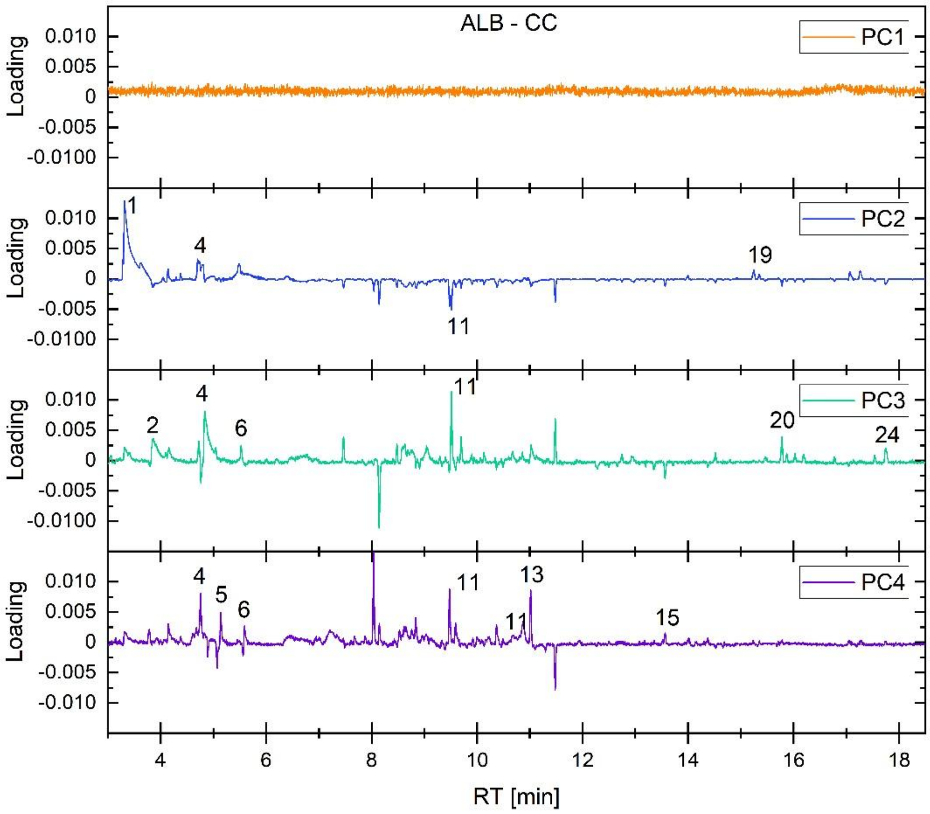

Figure S5. PCA - Loading plots of ALB - CC: Orange: PC1, blue: PC2, green: PC3, purple: PC4. The noise of PC1 is due to the fact, that for CC only data of larvae and not infested tree were available. CC larvae emit less VOC than trees in general, so the signal to noise ratio of CC data is higher than in the other classes after normalization.

Figure S6: Principal Component Analysis (PCA) - Loading plots of the class comparison poplar long-horned beetle *Saperda carcharias* (SC) vs. goat moth *Cossus cossus* (CC)

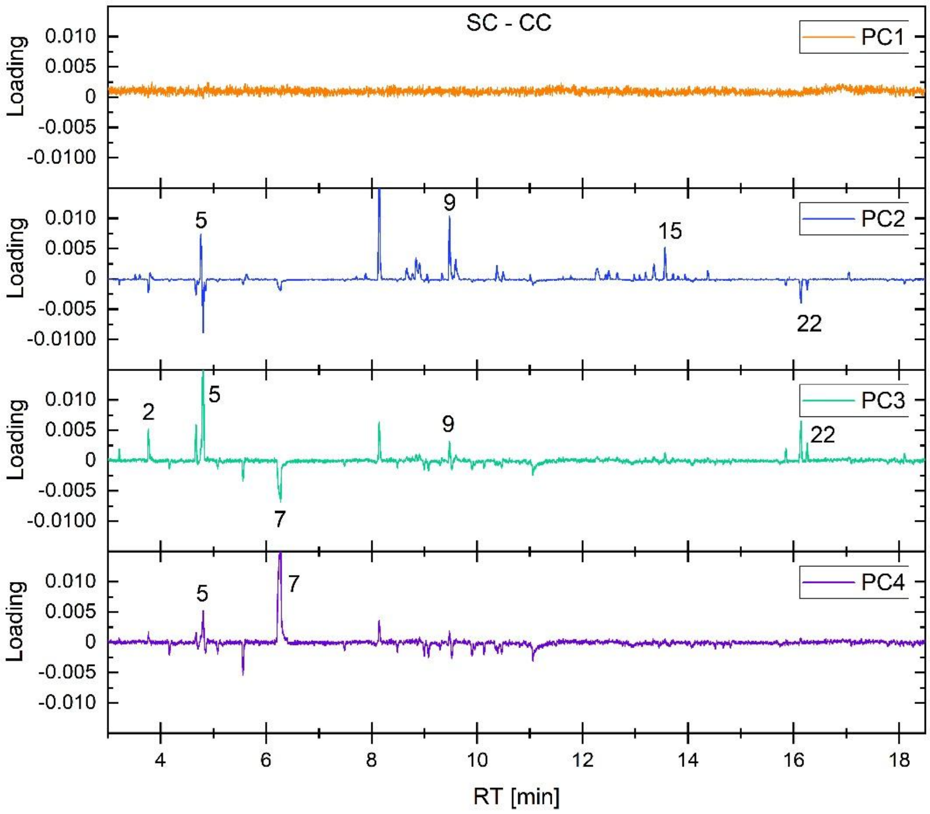

Figure S6. PCA - Loading plots of SC - CC: Orange: PC1, blue: PC2, green: PC3, purple: PC4. The noise of PC1 is due to the fact, that for CC only data of larvae and not infested tree were available. CC larvae emit less VOC than trees in general, so the signal to noise ratio of CC data is higher than in the other classes after normalization.

Figure S7: 2D - Linear Discriminant Analysis (LDA) - Plot of the comparison of Asian longhorned beetle *Anoplophora glabripennis* (ALB) vs. poplar long-horned beetle *Saperda carcharias* (SC) vs. goat moth *Cossus cossus* (CC)

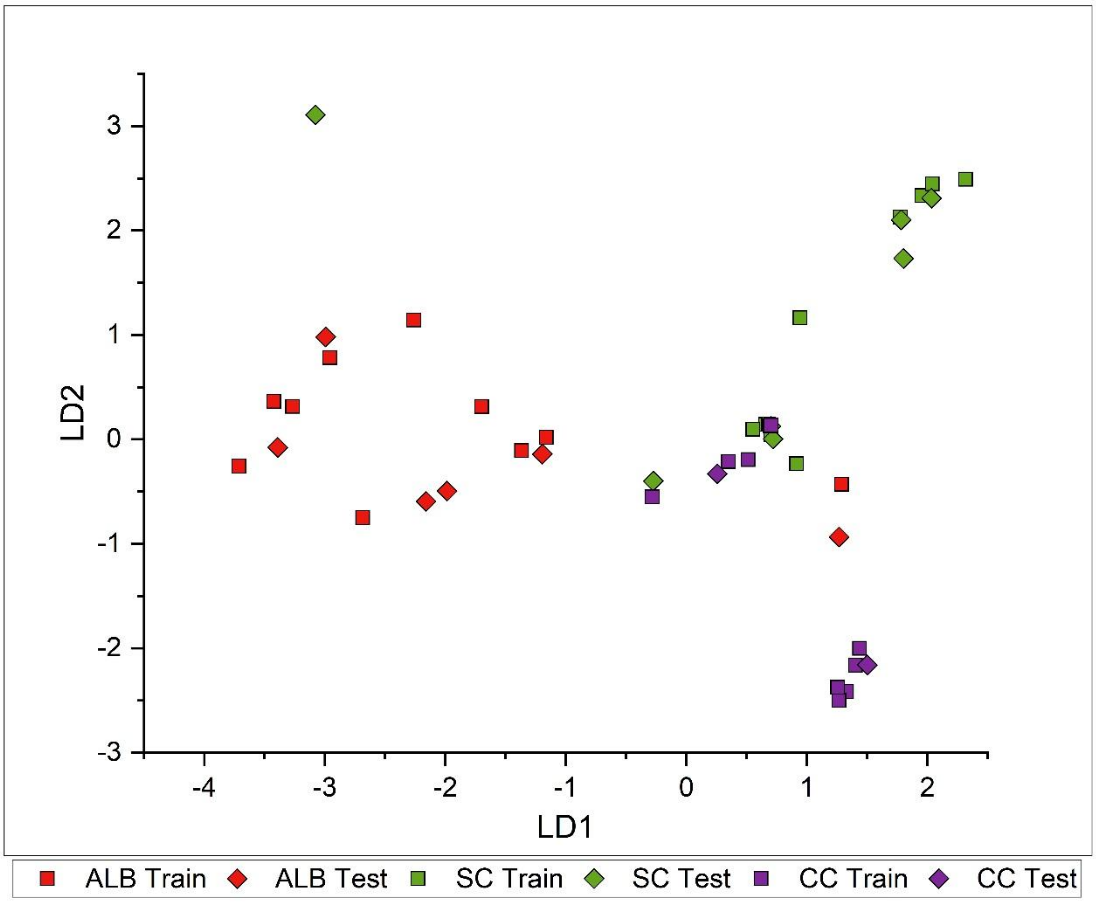

Figure S7. 2D-LDA-Plot of ALB – SC – CC: Red: ALB, green: SC, purple: CC, ■: Train data; ♦: Test data. “Leave one out” crossvalidation score: 87 %, “k-fold” cross validation score: 80 %, accuracy ALB: 83 %, accuracy SC: 71%, accuracy CC: 100 %. It can be seen that via LD1 the native beetles SC and CC can be differentiated from ALB, and that via LD2 SC and CC can be separated from each other.

Figure S8: 3D - Linear Discriminant Analysis (LDA) - Plot of the comparison of Asian longhorned beetle *Anoplophora glabripennis* (ALB) vs. Healthy tree (HT) vs. poplar long-horned beetle *Saperda carcharias* (SC) vs. goat moth *Cossus cossus* (CC)

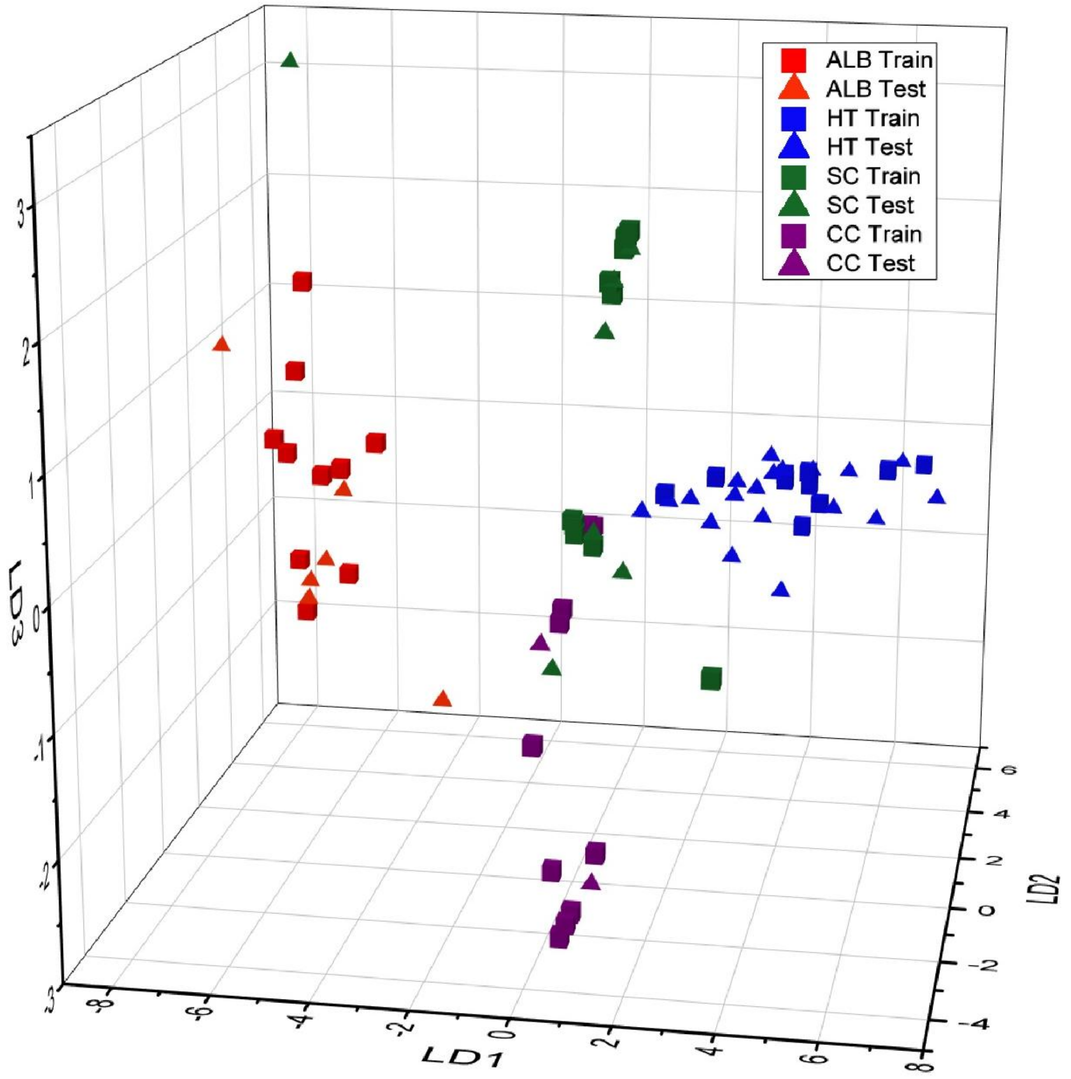

Figure S8. 3D-LDA-Plot of ALB – HT- SC – CC: Red: ALB, blue: HT, green: SC, purple: CC, ■: Train data; ▲: Test data. “Leave one out” crossvalidation score: 93 %, “k-fold” cross validation score: 75 %, accuracy ALB: 83 %, accuracy HT: 89 %, accuracy SC: 71 %, accuracy CC: 100 %. It can be seen that via LD1 ALB can be separated from HT and the native beetles SC and CC, that via LD2 ALB can be differentiated from SC and CC, and that via LD3 SC and CC can be separated from each other.

Figure S9: Principal Component (PCA) - 2D - Score plots of the comparison of Asian longhorned beetle *Anoplophora glabripennis* (ALB) vs. Healthy tree (HT)

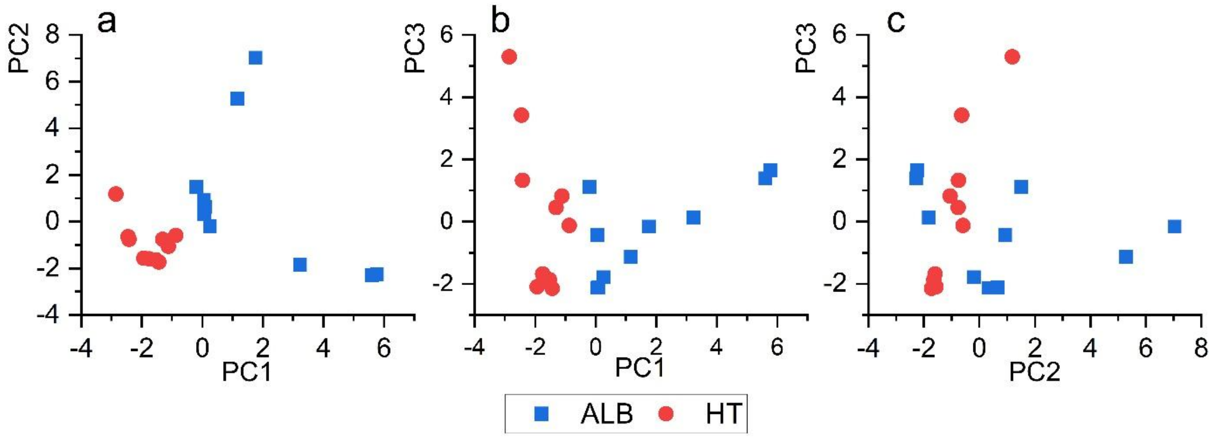

Figure S9. PCA 2D-Score-Plots ALB -HT. a: PC1 vs. PC2, b: PC1 vs. PC3, c: PC2 vs. PC3, blue: ALB, red: HT.

Figure S10: Principal Component (PCA) - 2D - Score plots of the comparison of poplar long-horned beetle *Saperda carcharias* (SC) vs. Healthy tree (HT)

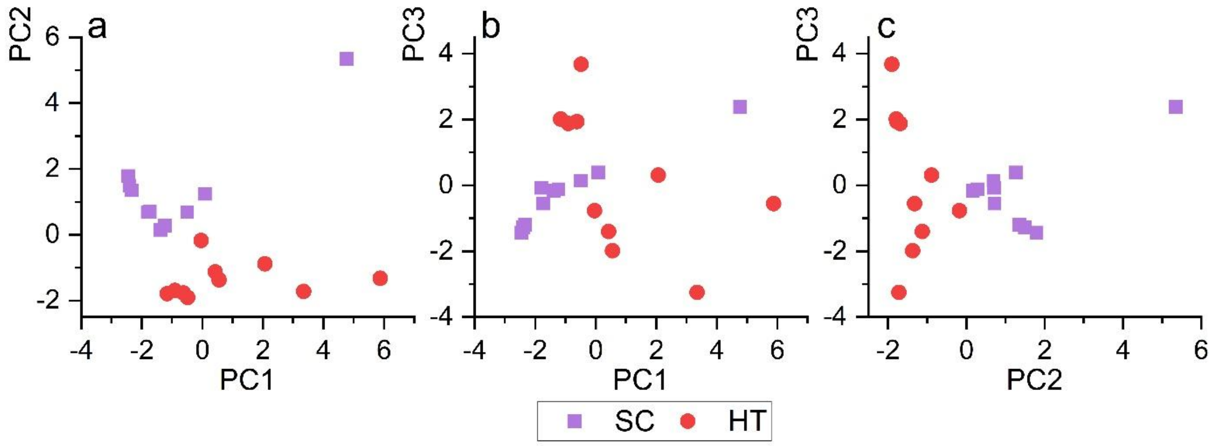

Figure S10. PCA 2D-Score-Plots SC -HT. a: PC1 vs. PC2, b: PC1 vs. PC3, c: PC2 vs. PC3, purple: SC, red: HT.

Figure S11: Principal Component (PCA) - 2D - Score plots of the comparison of goat moth *Cossus cossus* (CC) vs. Healthy tree (HT)

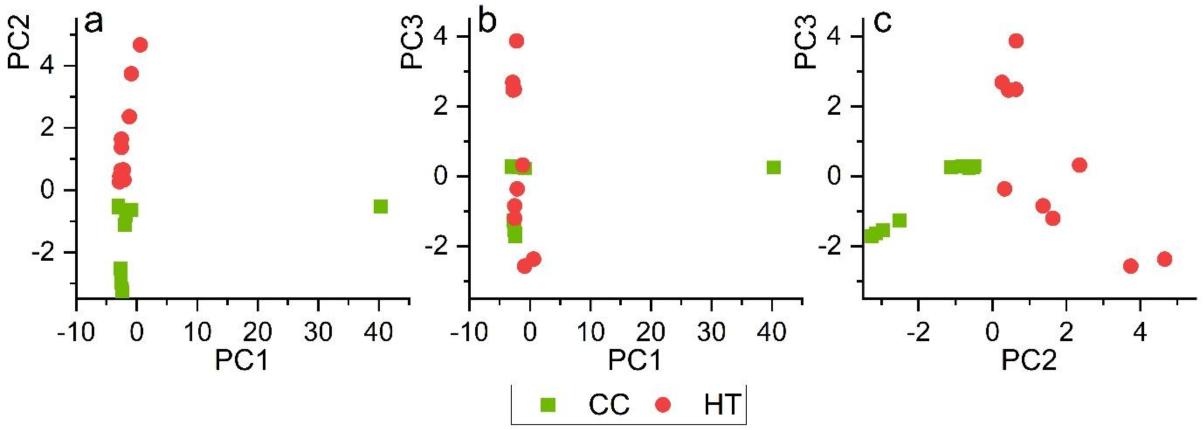

Figure S11. PCA 2D-Score-Plots CC -HT. a: PC1 vs. PC2, b: PC1 vs. PC3, c: PC2 vs. PC3, green: CC, red: HT.

Figure S12: Principal Component (PCA) - 2D - Score plots of the comparison of Asian longhorned beetle *Anoplophora glabripennis* (ALB) vs. poplar long-horned beetle *Saperda carcharias* (SC)

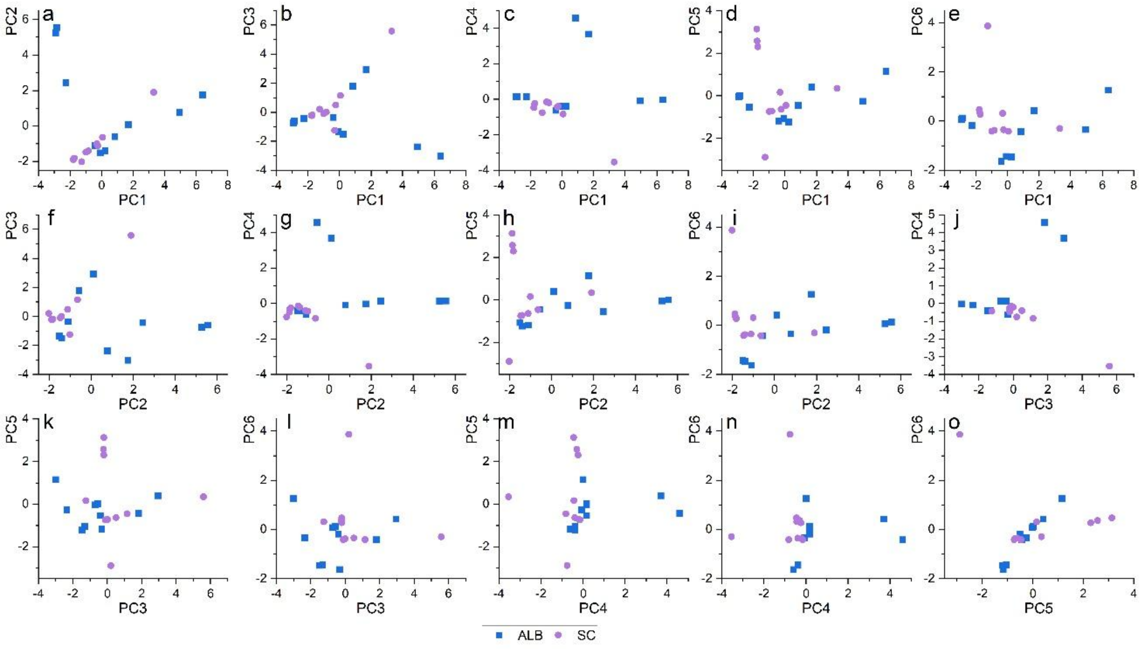

Figure S12. PCA 2D-Score-Plots ALB -SC. a: PC1 vs. PC2, b: PC1 vs. PC3, c: PC1 vs. PC4, d: PC1 vs. PC5, e: PC1 vs. PC6, f: PC2 vs. PC3, g: PC2 vs. PC4, h: PC2 vs. PC5, i: PC2 vs. PC6, j: PC3 vs. PC4, k: PC3 vs. PC5, l: PC3 vs. PC6, m: PC4 vs. PC5, n: PC4 vs. PC6, o: PC5 vs. PC6, blue: ALB, purple: SC.

Figure S13: Principal Component (PCA) - 2D - Score plots of the comparison of Asian longhorned beetle *Anoplophora glabripennis* (ALB) vs. goat moth *Cossus cossus* (CC)

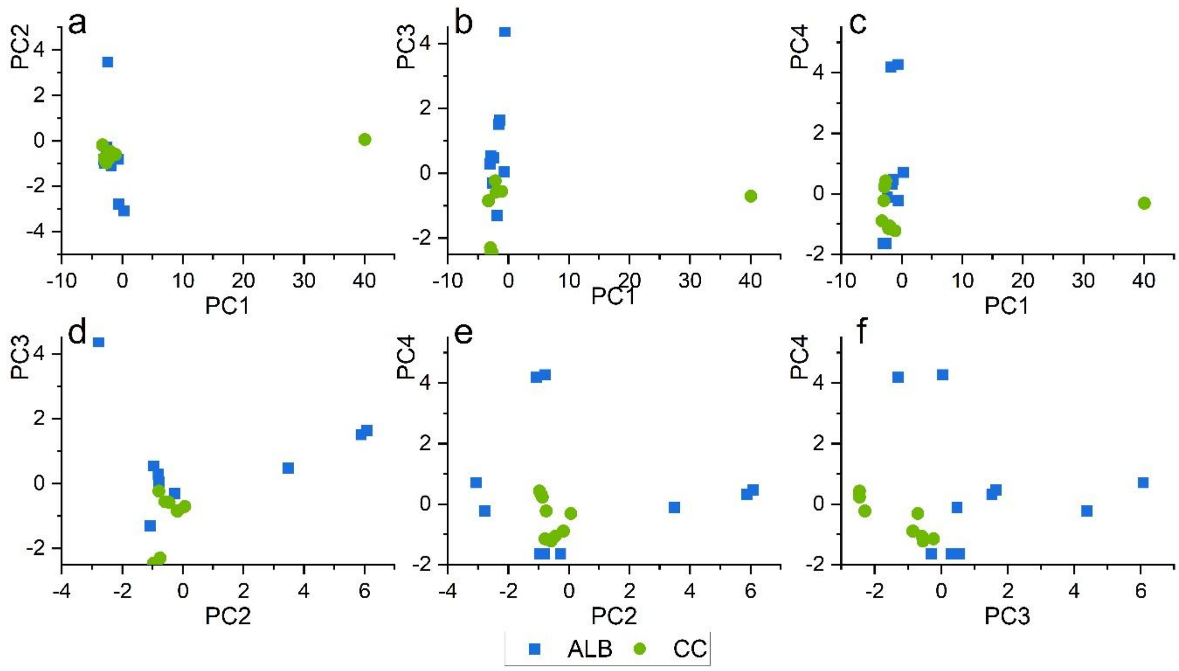

Figure S13. PCA 2D-Score-Plots ALB - CC. a: PC1 vs. PC2, b: PC1 vs. PC3, c: PC1 vs. PC4, d: PC2 vs. PC3, e: PC2 vs. PC4, f: PC3 vs. PC4, blue: ALB, green: CC.

Figure S14: Principal Component (PCA) - 2D - Score plots of the comparison of poplar long-horned beetle *Saperda carcharias* (SC) vs. goat moth *Cossus cossus* (CC)

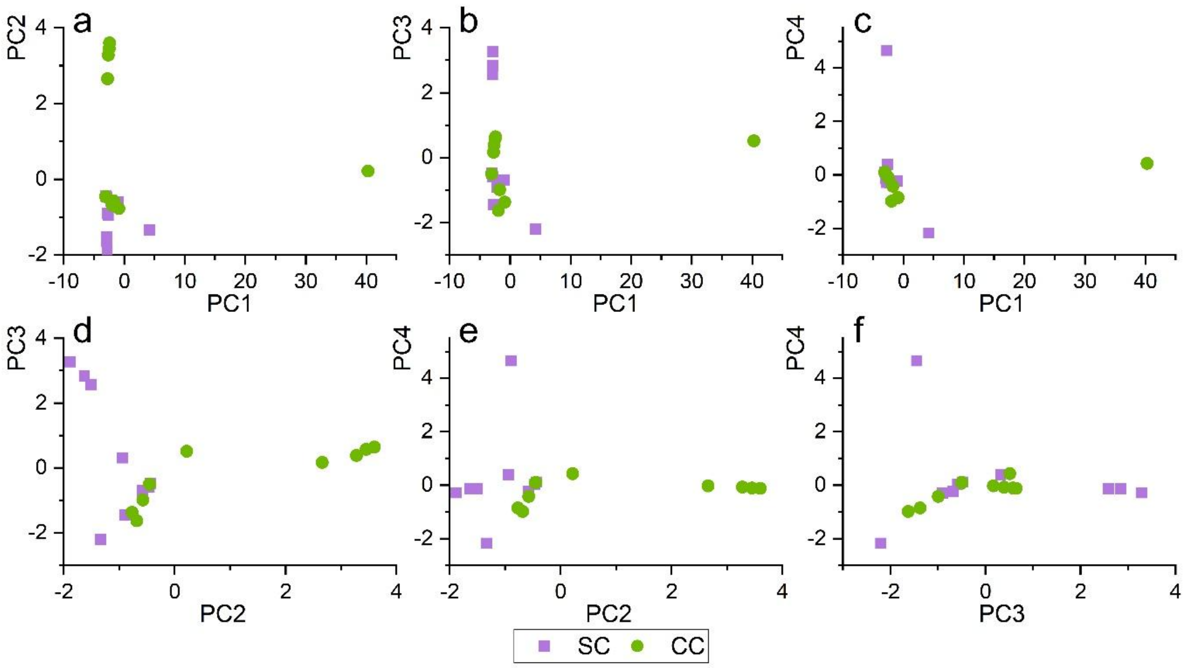

Figure S14. PCA 2D-Score-Plots SC - CC. a: PC1 vs. PC2, b: PC1 vs. PC3, c: PC1 vs. PC4, d: PC2 vs. PC3, e: PC2 vs. PC4, f: PC3 vs. PC4, purple: SC, green: CC.
